# Measurement properties of tools used to assess depression in adults with and without autism spectrum conditions: A systematic review

**DOI:** 10.1002/aur.1922

**Published:** 2018-01-23

**Authors:** S. A. Cassidy, L. Bradley, E. Bowen, S. Wigham, J. Rodgers

**Affiliations:** ^1^ School of Psychology University of Nottingham UK; ^2^ Institute of Neuroscience, Newcastle University UK; ^3^ Centre for Innovative Research Across the Life Course Coventry University UK; ^4^ Centre for Violence Prevention University of Worcester UK

**Keywords:** autism spectrum condition, general population, Asperger syndrome, depression, measurement properties, assessment, systematic review, COSMIN

## Abstract

Depression is the most commonly experienced mental health condition in adults with autism spectrum conditions (ASC). However, it is unclear what tools are currently being used to assess depression in ASC, or whether tools need to be adapted for this group. This systematic review therefore aimed to identify tools used to assess depression in adults with and without ASC, and then evaluate these tools for their appropriateness and measurement properties. Medline, PsychINFO and Web of Knowledge were searched for studies of depression in: (a) adults with ASC, without co‐morbid intellectual disability; and (b) adults from the general population without co‐morbid conditions. Articles examining the measurement properties of these tools were then searched for using a methodological filter in PubMed, and the quality of the evidence was evaluated using the COSMIN checklist. Twelve articles were identified which utilized three tools to assess depression in adults with ASC, but only one article which assessed the measurement properties of one of these tools was identified and thus evaluated. Sixty‐four articles were identified which utilized five tools to assess depression in general population adults, and fourteen articles had assessed the measurement properties of these tools. Overall, two tools were found to be robust in their measurement properties in the general population—the Beck Depression Inventory (BDI‐II), and the patient health questionnaire (PHQ‐9). Crucially only one study was identified from the COSMIN search, which showed weak evidence in support of the measurement properties of the BDI‐II in an ASC sample. Implications for effective measurement of depression in ASC are discussed. ***Autism Res*** *2018, 11: 738–754*. © 2018 The Authors Autism Research published by International Society for Autism Research and Wiley Periodicals, Inc.

**Lay Summary:**

Depression is the most common mental health problem experienced by adults with autism. However, the current study found very limited evidence regarding how useful tools developed for the general population are for adults with autism. We therefore suggest how these tools could be adapted to more effectively assess depression in adults with autism, and improve these individuals access to mental health assessment and support.

## Introduction

Recent research has shown that a majority of adults with an autism spectrum condition (ASC) meet diagnostic criteria for at least one psychiatric condition (79%), with depression being the most common [Lever & Geurts, 2016]. Studies have also highlighted high rates of depression in adults with ASC [e.g., Cassidy et al., 2014; Hofvander et al., 2009]. Depression is also the most common first concern prior to adults obtaining an ASC diagnosis [Jones, Goddard, Hill, Henry, & Crane, 2014]. Adults with ASC, without co‐morbid intellectual disability (the focus of this review), who have greater insight into their own difficulties, are also more likely to experience depression than those with less insight [Gotham, Bishop, Brunwasser, & Lord, 2014]. The consequences of not detecting depression in those with ASC can be devastating. Depression has been shown to increase risk of adults with ASC contemplating, planning [Cassidy et al., 2014], or dying by suicide [Hirvikoski et al., 2016]. Clearly, it is crucial to effectively identify depression in this group. However, it is unclear if there are valid tools available to assess depression in those with ASC.

ASC is characterized by difficulties in socialization, imagination, communication, narrow obsessive interests, and sensory difficulties [APA, 2013]. A number of depressive symptoms may overlap with symptoms and behaviors in ASC, such as social withdrawal, difficulties with sleep, flat affect, and reduced eye contact [Stewart, Barnard, & Pearson, 2006], resulting in diagnostic overshadowing. This overlap of symptoms could present challenges for clinicians in the identification of depression in ASC, particularly when using tools developed for those without ASC. For example, individuals with ASC could score higher on traditional depression measures, by endorsing items which may be capturing their ASC symptoms as opposed to depression per se. This could mean that depression symptoms are over‐endorsed using these measures, even in the absence of depression, in those with ASC compared to those without ASC. Equally, lack of autism‐specific items tailored to the unique presentation of depression in ASC, such as loss of absorption in a special interest [Clarke, Littlejohns, Corbett, & Joseph, 1989; Gillberg, 1985], agitation, change in sleep pattern or social withdrawal [Ghaziuddin, 2005], could potentially underestimate depression in this group. Either way, there are reasons to suspect that traditional measures of depression may not adequately capture the unique presentation of depression in those with ASC, and therefore increase the risk of inaccurate identification of depression in these individuals.

In addition to the overlap of symptoms between ASC and depression, there are cognitive characteristics of ASC which could affect the diagnostic accuracy of existing depression measures in this group. For example, many depression measures rely on self‐report, and the ability to reflect and report on one's internal emotional experience. Difficulties articulating one's own internal emotional experience (termed Alexithymia) is common in those with ASC [e.g., Bird et al., 2010]. Literal interpretation of questions [Happé, 1995] could also impede ability to correctly interpret questions such as “I could not shake off the blues” [item 2 of the Centre for Epidemiological Studies Depression Scale—Revised; Eaton, Smith, Ybarra, Muntaner, & Tien, 2004]. Therefore, the mode of assessment and language used could affect the content validity of traditional depression measures in those with ASC.

Given the potentially tragic consequences of failing to accurately identify depression in adults with ASC, it is extremely important for clinicians, researchers and service providers to have access to appropriate tools to effectively assess depression in these individuals. However, it is unknown which tools to assess depression have been used in those with ASC, what the evidence is regarding the appropriateness and measurement properties of these tools, or whether existing tools used in the general population need to be adapted for those with ASC. To address this knowledge gap, we conducted a comprehensive two‐stage systematic review of the available evidence. First, we searched the literature for all available studies which have utilized a tool to assess depression frequently (at least twice), with evidence of validity (i.e., with reference to a published study), in; (a) adults with ASC, without co‐morbid intellectual disability; and (b) adults from the general population, without co‐morbid conditions.

To guarantee appropriate conclusions regarding the appropriateness and measurement properties of an instrument, it is crucial to ascertain whether studies are of high methodological quality. To accomplish this, a checklist was developed in a multi‐disciplinary, international consensus‐study involving 43 experts in health outcome measurement: The consensus based standards for the selection of health measurement instruments (COSMIN). A growing number of studies are using COSMIN to assess the evidence for the appropriateness and measurement properties of tools in ASC [Hanratty et al., 2015; Wigham & McConachie, 2014], in order to make evidence based recommendations for future research and clinical practice. Hence, we searched for evidence regarding the measurement properties of these tools in each group, using a comprehensive search tool validated for this purpose [Mokkink et al., 2010; Terwee, Jansma, Riphagen, & de Vet, 2009], and subsequently rated the quality of the available evidence using this validated research tool [COSMIN, Mokkink, Terwee, Patrick, Alonso, & Stratford, 2012]. Including searches looking at both adults with and without ASC enables us to draw comparisons in the state of the evidence between each group, and given the paucity of literature exploring depression in ASC, identify robust candidate measures used in the general population which could be successfully adapted for adults with ASC. From this synthesis of the available evidence, we subsequently make recommendations for future research aiming to effectively assess depression in ASC.

## Review Methods: Stage 1

The protocol for this review is registered within the International Register of Systematic Reviews (Registration number: CRD42016035220), and can be accessed online (http://www.crd.york.ac.uk/PROSPERO/prospero.asp). This systematic review follows the guidelines for Preferred Reporting Items for Systematic Reviews and Meta‐Analyses standards.

### Search Strategy

The following electronic bibliographic databases were searched: Medline, PsychINFO and Web of Knowledge. The Cochrane library was also searched to confirm that no other systematic reviews of the current study topic existed. There were two searches carried out in stage one for depression measures used in; (a) adults with ASC, without co‐morbid intellectual disability; and (b) general population adults, without any co‐morbid conditions. The terms for each search strategy are included in Table 1. The searches were restricted to peer‐reviewed articles published in the English language, between 1992 and June 2016—when the last searches were run. The current study focused on literature pertaining to ASC without co‐morbid intellectual disability, which is frequently referred to as Asperger syndrome (AS). AS was first included as a separate diagnosis in the WHO International Classification of Diseases (ICD‐10) in 1992, so we focused on studies published after this date, when we expected reference to AS to be more consistent in the literature.

**Table 1:** Stage 1 Review Search Terms

1. (ASC or ASD or Asperg* or Autis* or high functioning or pervasive developmental disorder* or PDD or HFA)
2. (general population or population sample or community sample or national* survey or household* survey or nonreferred or nonclinical or population screen*)
3. (adult*)
4. (assess* or tool or treatment outcome or measure* or scale or quotient or inventory or instrument)
5. (depress* or low mood or affective disorder or mood disorder)
6. randomised controlled trial or randomized controlled trial
7. random*
8. comparative stud*
9. prospective stud*
10. intervention
11. treatment effectiveness evaluation or treatment response or treatment study
12. epidemiolog*
13. prevalence
14. (General Population Search) (6 or 7 or 8 or 9 or 10 or 11 or 12 or 13) and (2 and 3 and 4 and 5)
15. (ASC Search) (6 or 7 or 8 or 9 or 10 or 11 or 12 or 13) and 1 and 3 and 4 and 5
16. limit 14 and 15 to English Language; 1992—current; age 18 years +

### Selection Criteria

COSMIN recommends focusing searches on a well‐defined group and outcome. Therefore, studies had to focus on a tool to specifically assess depression (as opposed to a subscale contained within a larger measure, e.g., structured clinical interview [SCID]), clinically defined as in the ICD‐10, and diagnostic and statistical manual of mental disorders (DSM‐V). We searched for studies utilizing tools to assess either the prevalence of depression (epidemiological/population studies), or to assess outcomes (treatment/intervention and longitudinal/cohort studies). To be included, studies had to focus on adults aged 18 years and over, without co‐morbid intellectual disability. Where the age range was partly outside this, studies were included if 50% or more of the total sample studied was over 18 years old, and the mean age of the sample was 18 years or above. This ensured that the tools were likely to be appropriate for adults. We excluded articles using tools which had been adapted specifically for a population other than ASC or general population (e.g., for older adults, a particular gender, or a specific culture). In search one, which included studies focusing on the adult general (nonclinical) population, studies were included if data from the general population were presented separately, and the sample consisted of 50% or more participants from the general population. Any studies including an ASC comparison group were excluded at this stage and considered for inclusion in search two, which included studies focusing on adults with ASC without co‐morbid intellectual disability. If a study included participants with a range of different diagnoses, the study was included if ASC data were presented separately, and if 50% or more participants had ASC. In searches where studies had used more than one version of a tool, we included studies using the most up to date version (e.g., the BDI‐II).

One reviewer (L.B.) screened the titles and abstracts of articles for inclusion, and where there was any doubt on whether an article should be carried over to the full text sift, it was included. L.B. then conducted the full text sift of articles, with any ambiguous papers discussed with S.C., E.B., and J.R. to reach consensus. All references of included articles were also searched for additional articles to include.

### Data Extraction

Data extraction was performed by L.B., and 20% of articles independently checked by S.C. A data extraction form was adapted from a form used in similar research previously [Wigham & McConachie, 2014]. Data pertaining to: participant characteristics, tools used, domains captured and study type were recorded.

## Results: Stage 1

### ASC

**Figure 1:**
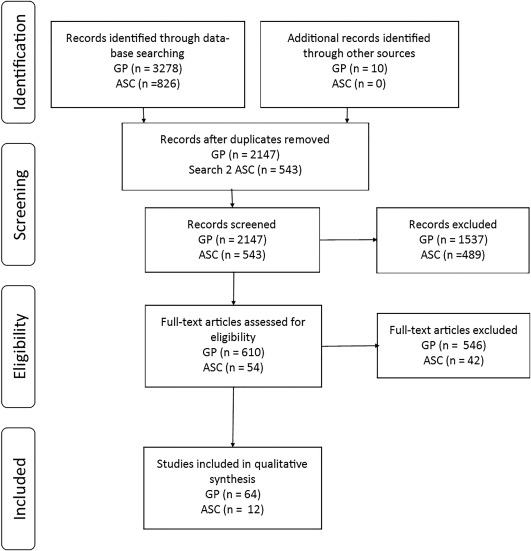
Results of search one.

The search for studies using tools to assess depression in adults with ASC, without co‐morbid intellectual disability, identified 543 articles which were screened, and 12 of these were retained for analysis (Fig. 1). Seven of these studies were cross‐sectional studies characterizing mental health in those with ASC, including between 27 and 255 participants with ASC. Five of these studies used tools to assess depression after a treatment trial, including one (in a single case study of CBT) to 49 participants. Taken together, the evidence shows a small number of cross sectional and treatment outcome studies which have used tools to assess depression which have some evidence of validity (but not in the sample studied). It is also important to note that none of the tools utilized in these studies had been developed or validated for people with ASC.

Four different tools were used to assess depression in the studies (Table 2). Three of these had also been used in the general population (BDI‐II, HRSD, PHQ‐9), but one had not been identified in the general population search: the Montgomery Asberg Depression Rating Scale (MADRS). Therefore, this measure was also considered further in stage 2.

**Table 2:** Characteristics of Depression Tools Identified in Search One

Measure (inc. version, where applicable, & date of publication	Aim of tool	Number of items (Subscales)	Response options (e.g., 4‐point scale, yes/no etc.)	Format (e.g., self report questionnaire, interview etc.)	Used in which references?	
					ASC	General Population
Beck Depression Inventory (BDI) Version II (1996)	Measure symptoms of depression	21	4 point scale	Self‐report questionnaire	Moss, Howlin, Savage, Bolton, and Rutter [2015], Gotham et al. [2014], Hillier, Fish, Siegel, and Beversdorf [2011], Limoges, Mottron, Bolduc, Berthiaume, and Godbout [2005], Cardaciotto and Herbert [2005], Cederlund, Hagberg, and Gillberg [2010]	Linda, Marroquín, and Miranda, (2012), Spitzer et al. (2012), Yun et al. (2015), Leiderman, Lolich, Vázquez, and Baldessarini, (2012), Spitzer et al. (2013), Koivumaa‐Honkanen, Kaprio, Honkanen, Viinamäki, and Koskenvuo, (2004), Joo et al. (2009)
Hamilton rating scale for depression (HRSD) (1960)	Measure of depressive symptoms in diagnosed patients to assess treatment outcomes	21 (depressive mood, guilt, and suicidal tendencies)	Range from 3 to 5‐point ratings scales	Clinician administered interview	Buchsbaum et al. [2001]	Khedr et al. [2013], Zdanowicz, Lepiece, Tordeurs, Jacques, and Reynaert [2004], Carta et al. [2011)]
Montgomery Asberg Depression Rating Scale (MADRS) (1979)	Measure of depressive symptoms in diagnosed patients to assess treatment outcomes	10 (Apparent sadness, Reported sadness, Inner tension, Reduced sleep, Reduced appetite, Concentration difficulties, Lassitude, Inability to feel, Pessimistic thoughts, Suicidal thoughts)	6‐point scale	Clinician administered interview	Wentz, Nydén, and Krevers [2012], Rydén and Bejerot [2008]	N/A
Centre for Epidemiological Studies Depression Scale (CESD) (1977)	Measure depressive symptoms in the general population.	20	4 point scale	Self‐report questionnaire	N/A	Shanahan et al. [2015], Oyama and Sakashita [2014], Lee, Lee, and Kim [2015], Steinhausen, Gundelfinger, and Metzke [2009], Furihata et al. [2011], Oh et al. [2013], Aaro, Herbec, Bjorngaard, Manczuk, and Zatonski [2011], Nomura, Inoue, Kusumi, Uemura, and Nakashima [2008], Sousa, Zauszniewski, and Ala'a [2010], Carroll, Cassidy, and Côté [2000, 2003, 2004], Rowan, Davidson, Campbell, Dobrez, and MacLean [2002], Abravanel and Sinha [2015], Li, Dai, Ekperi, Dehal, and Zhang [2011], Ciarlo, Shern, Tweed, Kirkpatrick, and Sachs‐Ericsson [1992], Smith, Marcus, Lewis, Fitzgibbon, and Schreiner [1998], Kear [2002], Yan et al. [2003], Schulz et al. [2008], Borders, Guillén, and Meyer [2014], Pargament, Magyar, Benore, and Mahoney [2005], Pedlow and Niemeier [2013], Gorka, Ali, and Daughters [2012], Lee and Seo [2013], Yaroslavsky, Pettit, Lewinsohn, Seeley, and Roberts [2013], Polak, Houghton, Reeder, Harper, and Conner [2014], Rosenberg et al. [2010], Hincapié, Cassidy, and Côté [2008], Donald and Dower [2002], Muntaner and Barnett [2000], Moscato et al. [1997], Côté, Cassidy, and Carroll [2000], Stewart et al. [2003]
Human Population Laboratory Depression Scale (HPL) (1981)	Measure of depressive symptoms	18	True/False responses	Self‐ report questionnaire	N/A	Everson, Roberts, Goldberg, and Kaplan [1998]
Major Depression Inventory (MDI) (2001)	Measure of depression symptoms in the general population and as a diagnostic measure of major or moderate depression	10	6 point scale	Self‐ report questionnaire	N/A	Andersen, Thielen, Bech, Nygaard, and Diderichsen [2011]
Patient Health Questionnaire (PHQ) (9 item: 2002; 2 item: 2010).	Depression screening in primary care settings	2 (first 2 items of PHQ‐9) 9	4 point scale	Self‐ report questionnaire	Fortuna et al. [2016], Morgan, Leatzow, Clark, and Siller [2014], Mazurek [2014]	Li, Ford, Zhao, Tsai, and Balluz [2012], Cockayne et al. [2011], Anno et al. [2015], Eisenberg, Gollust, Golberstein, and Hefner [2007], Maske et al. [2016], Beydoun, Shroff, Beydoun, and Zonderman [2010], Lê Cook et al. [2014], 6617, Shim, Baltrus, Ye, and Rust [2011]
Short depression‐happiness scale (SDHS) (2004)	Measure of depression and happiness through a continuum in research and clinical practise	6	4 point scale	Self‐ report questionnaire	N/A	Kupeli et al. [2015]
Zung Self Rating Depression Scale (ZSDS) (1965)	Measure of depressive symptoms in the general population	20	4 point scale	Self‐ report questionnaire	N/A	Pardini et al. [2016], Yannakoulia et al. [2007], Pitsavos, Panagiotakos, Lentzas, and Stefanadis [2005]

## General Population

The search for studies using depression tools in general population adults without co‐morbid conditions identified 2,147 articles which were screened, 64 of which were retained for analysis (Fig. 1). Most of the included studies reported results from large cross‐sectional or longitudinal studies of the population, including tools measuring the prevalence of depression, and/or change in depression in response to treatment programs. The sample size of the included studies ranged from 10 to 133,113. Taken together the evidence shows a number of large scale population studies which have used tools, with evidence of validity, to assess depression in the general population without co‐morbid conditions.

Eight different tools were used to assess depression in the studies (Table 2). Self‐report questionnaires included: The Beck Depression Inventory, second edition [BDI‐II, Beck, Steer, & Brown, 1996]; The Centre for Epidemiological Studies Depression Scale [CESD, Radloff, 1977]; The Human Population Laboratory Depression Scale [HPL; Kaplan, Roberts, Camacho, & Coyne, 1987]; the Major Depression Inventory [MDI; Bech, 1997]; the patient health questionnaire, 2 and 9 item versions [PHQ‐2/9; Kroenke & Spitzer, 2002]; the Short Depression‐Happiness Scale [SDHS; Joseph, Linley, Harwood, Lewis, & McCollam, 2004]; and the Zung Self Rating Depression Scale [ZDS; Zung, 1965]. A clinician administered interview, the Hamilton Rating Scale for Depression [HRSD; Hamilton, 1960] was also identified. Three of these tools (HPL; MDI; SDHS) had each only been used in one study. Therefore, these tools were not considered further, as we were interested in tools which had been used frequently with evidence of validity (i.e., by reference to a previous validation study). The CESD has been revised to better map depression according to DSM‐V criteria [Eaton et al., 2004], but the up‐dated version was not identified in the initial searches (likely due to inclusion of suicidality items in the more recent version). Hence, we searched for studies which assessed the measurement properties of the CESD‐R, rather than the previous version of the tool, given that the revised version will need to be used in future research studies. Hence, five tools (BDI‐II, CESD‐R, PHQ‐9, ZDS, and HRSD) were considered further in stage 2 (Table 1).

### Summary

Six tools which had been used frequently (at least twice) with evidence of validity in adults from the general or ASC populations were identified from the searches and were considered further. In comparison to the general population, there were relatively few studies which utilized validated tools to assess depression in adults with ASC (12 vs. 64), and none which used a tool developed or validated specifically for adults with ASC (Table 1).

## Review Methods: Stage 2

The second stage of the review searched for evidence of the measurement properties of the tools identified in stage 1. To do this, a comprehensive search was carried out using a methodological filter in PubMed, designed to search for studies assessing the measurement properties of health outcome assessment tools [Terwee et al., 2009]. As in stage 1, we focused on studies which had explored the measurement properties of the tools in: (a) adults with ASC, without co‐morbid intellectual disability; and (b) general population adults, without co‐morbid conditions. In the case of general population adults, without co‐morbid conditions, again we excluded articles which explored the measurement properties of measures in nonrepresentative samples, such as patients, college students, older adults or other subgroups (e.g., single gender, LGBT, army veterans). This was to focus on evidence for the measurement properties of tools used in representative general population samples, as opposed to nonrepresentative sub‐groups of the population.

### Data Extraction Method

Once articles were identified from the search, the methodological quality of each article was assessed using the COSMIN checklist (Consensus based Standards for the selection of health based measurement Instruments) [Mokkink et al., 2012]. COSMIN rates the evidence in support of nine measurement properties on a 4‐point scale (from excellent to poor): internal consistency, reliability, measurement error, content validity, structural validity, hypothesis testing, criterion validity, responsiveness to change, and cross‐cultural validity. COSMIN implements a ‘worst score counts’ method, by which an overall rating is assigned to each measurement property based on the lowest score provided. For example, if a study is rated excellent on all criteria related to internal consistency (e.g., Cohen's Kappa was calculated, an adequate sample size was utilized etc.), but the study failed to check the uni‐dimensionality of the scale, then this study would still be rated as ‘poor’ overall [Mokkink et al., 2010]. The checklists were completed by SC, with 20% of the articles rated by SW, who was trained and experienced in using COSMIN. Percentage agreement between SC and SW was 72%, similar to previous studies [e.g., Wigham & McConachie, 2014]. Disagreements were resolved with discussion and these agreed ratings were utilized in the subsequent evidence synthesis.

### Evidence Synthesis

The quality of the evidence in support of each measurement property needs to be considered in the context of the studies’ findings to gauge the amount of evidence available for or against each measurement property. First, the quantitative findings from each study are given a rating of positive (in support of the property), indeterminate (not possible to deduce whether the evidence is for or against the property), or negative (evidence against the property). For example, criterion validity is considered positive when the study supplies convincing evidence that the criterion used is indeed a gold standard, and the correlation between the outcome measure and the gold standard criterion is greater than 0.7 [De Vet, Terwee, Mokkink, & Knol, 2011]. Subsequently, the quality of the evidence is considered in the context of the studies’ quantitative findings. *Strong* evidence (+++/−−) is defined as one methodologically excellent or several good studies which find consistent evidence for or against a measurement property; *moderate* evidence (++/−) is defined as one methodologically good or several fair studies which find consistent evidence for or against a measurement property; *limited* evidence (+/−) is defined as one methodological fair study finding evidence for or against a measurement property; *conflicting* evidence (+/−) is where the evidence for or against a measurement property is not consistent between studies; and *indeterminate* evidence (?), is where there are only studies of poor methodological quality available for a measurement property [Mokkink et al., 2012].

## Results: Stage 2

### Autism Spectrum Conditions

**Figure 2:**
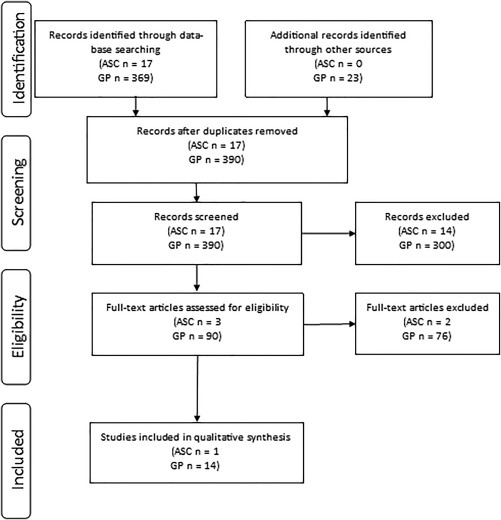
Results of search two.

The PubMed search for studies assessing the measurement properties of depression tools used in adults with ASC, without co‐morbid intellectual disability, identified 17 articles which were screened, one of which was retained for analysis (Fig. 2). This study assessed the measurement properties of the BDI‐II in adults with ASC (Table 3). There was indeterminate evidence for internal consistency, as alphas were not calculated for each subscale, and the uni‐dimensionality of the instrument was not checked in the current sample. However, there was a high Cronbach's alpha (.87) for the whole scale. There was weak evidence in support of hypothesis testing, as hypotheses were not formulated, but it was possible to deduce what was expected, and adequate correlations were reported with other measures of similar constructs (*r* > .63). There was weak evidence for criterion validity, as it was not clear how missing items were handled, despite acceptable correlations with clinical diagnosis of depression (*r* = .8).

**Table 3:** Characteristics of the Study Populations Included in the Stage Two Evidence Synthesis

Tool	Group		Article	Study population	Study type (prospective, case‐control etc.)	Mean age (SD) years; range	N	Male n, female n.	Country
	ASC	GP							
BDI‐II	X		Gotham et al. [2015]	Adults with ASC, without co‐morbid learning disability	Case‐control	20.7 (3.9) 16–30 years	50	45 male, 5 female	US
BDI‐II		X	Abubakar et al. [2016]	Community sample and professionals 1	Cognitive interviews	Not reported	29	Not reported	Kenya
				Community sample 2	Population study		221	86.7% female	
				Community sample 3	Case‐control		29	80.1% female	
				Caregivers of adolescents with HIV4			77		
BDI‐II		X	Kjærgaard, Arfwedson Wang, Waterloo, and Jorde [2014]	Selected healthy sample	Population study	55.5 (10), 30–75 years	352	168 male, 189 female.	Norway
BDI‐II		X	Gomes‐Oliviera et al. [2012]	Medical students	Population study	24.6 (1.2), 22–26 years	60	51% female	Brazil
				General population adults		41 (10.8), 20–60 years	182	102 (56%) female	
BDI‐II		X	Segal, Coolidge, Cahill, and O'riley [2008]	Community dwelling a) older adults	Population study	a) 70.3, (SD 7.5), 55–90 years	147	42% male	US
				b) younger adults		b) 19.6, (SD 2.2), 17–29 years	229	36% female	
PHQ‐9		X	Chung et al. [2015]	MS	Longitudinal	51.81 (11.49)	1,603	301 male, 1,294 female	US
				SCI		40.39 (15.96)	3,694	2,836 male, 858 female	
				Community		46 (17.22)	3,000	1010 male, 1989 female	
PHQ‐9		X	Kiely and Butterworth [2015]	Community sample	Longitudinal	34.7, 32–37 years	546	58.5% female	Australia
						55, 52–58 years	1515	53.4% female	
PHQ‐9		X	Wang et al. [2014]	General population	Population study	47.1 (16.3) years	1045	366 male, 679 female	China
PHQ‐9		X	Kocalevent, Hinz, and Brähler [2013]	General population	Population study	48.9 (18.1)	5018	53.6% female	Germany
PHQ‐9		X	Yu, Tam, Wong, Lam, and Stewart [2012]	Community sample	Population study	>15 years	6028	2784 male, 3244 female	China
PHQ‐9		X	Martin, Rief, Klaiberg, and Braehler [2006]	General population	Population study	48.4 (18.1), 14–93 years	2066	53% female	Germany
ZDS		X	Yamazaki, Fukuhara, and Green [2005]	General Population	Population st udy	>16 years	3107	1573 male, 1534 female	Japan
ZDS		X	Chida, Okayama, Nishi, and Sakai [2004]	General Population	Population study	52,4 (16), 20–79 years	5547	2602 male, 2945 female	Japan
CESD‐R		X	Van Dam and Earleywine [2011]	Community sample	Cross‐sectional	30.6 (13.1)	7398	80.7% male	US
				Student sample		19.6 (1.8)	245	62% female	
MADRS		X	Schulte‐van Maaren et al. [2013]	General population	Cross‐sectional	40.3 (12.6)	1295	62.8% female	Netherlands
				Psychiatric outpatients		39.3 (12.3)	4627	61% female	

### General Population

The PubMed search for studies assessing the measurement properties of depression tools used in general population adults, without co‐morbid conditions, identified 390 articles which were screened, 14 of which were retained for analysis (Fig. 2). Table 3 shows the characteristics of the study populations included in the analysis.

The methodological quality of the included studies is presented in Table 4. None of the studies had explored measurement error or responsiveness to change—therefore these properties are not included in the table. The collated evidence pertaining to the measurement properties for each tool is presented in Table 5. Many of the articles reported data on differences in scores and normative data, which are important for interpretability [De Vet et al., 2011]. However, no studies reported minimal important change or floor or ceiling effects.

**Table 4:** Methodological Quality of Studies Included in Search Two Evidence Synthesis

Tool	Article	Internal Consistency	Reliability	Content Validity	Structural Validity	Hypothesis Testing	Cross cultural validity	Criterion Validity	Interpretability
BDI‐II	Gotham et al. [2015]	poor				fair		fair	
BDI‐II	Abubakar et al. [2016]	fair		excellent	fair	fair	poor		Y
BDI‐II	Kjærgaard et al. [2014]	poor				fair		good	Y
BDI‐II	Gomes‐Oliveira, Gorenstein, Neto, Andrade, and Wang [2012]	poor	fair			poor	fair	fair	Y
BDI‐II	Segal et al. [2008]	poor			fair	fair			Y
PHQ‐9	Chung et al. [2015]					fair			‐
PHQ‐9	Kiely and Butterworth [2015]							fair	Y
PHQ‐9	Wang et al. [2014]	fair	fair		fair	fair		fair	Y
PHQ‐9	Kocalevent et al. [2013]	fair			fair	fair			Y
PHQ‐9	Yu et al. [2012]	fair	fair		fair	fair			Y
PHQ‐9	Martin et al. [2006]					Good			Y
ZDS	Yamazaki et al. [2005]					fair			‐
ZDS	Chida et al. [2004]				excellent				Y
CESD‐R	Van Dam and Earleywine [2011]	excellent			excellent	good			Y
MADRS	Schulte‐van maaren et al. [2013]	poor						good	Y

**Table 5:** Quality of the Evidence of Each Tool from Search 2

Measure	Group		Measurement Properties							Interpretability
	ASC	GP	Internal Consistency	Reliability	Content Validity	Structural Validity	Hypothesis Testing	Criterion Validity	Cross‐cultural validity	Differences in scores between groups
BDI‐II	X		?				+	+		Y
		X	+	+	+++*	++	++	++	+	Y
PHQ‐9		X	++	++		++	++	++		Y
ZDS		X				+++	+			Y
CESD‐R		X	+++			+++	++			Y
MADRS		X	?					++		Y

*Content validity assessed in a translated version only.

? denotes unable to rate quality of evidence due to only evidence of poor quality being available.

***HDRS*.** No articles assessing the measurement properties of the HRDS in a general population sample without co‐morbid conditions were identified from the search.

***BDI‐II*.** Four studies were found to assess a range of measurement properties of the BDI‐II in general population adults, without co‐morbid conditions. There was weak evidence in support of internal consistency—many studies did not calculate Cronbach's alpha for each sub scale separately. However, all studies showed support for the internal consistency of the BDI‐II total score with acceptable alphas above (.7). There was weak evidence in support of test‐retest reliability with one fair study (as it was unclear how missing items were handled), with a high alpha (.89). There was strong evidence for content validity in one methodologically excellent study of the BDI‐II in a non‐English speaking Kenyan sample. There was moderate evidence in support of structural validity from two studies. Both studies showed fair evidence for a single factor solution. Evidence for hypothesis testing was moderate—the BDI‐II showed acceptable correlations with other depression measures (*r* > .57). There was weak evidence for cross‐cultural validity as there were weaknesses in the quality of the translations (only one forward/backward translation), or failure to pre‐test the items in a sample for interpretability and cultural relevance. There was moderate evidence for criterion validity. The BDI‐II showed adequate sensitivity (>.7) and specificity (>.8) in determining Major Depressive Episode with clinician ratings used as the criterion.

***PHQ‐9*.** Six studies were found which explored a range of measurement properties of the PHQ‐9 in general population adults. There was moderate evidence in support of internal consistency with adequate Cronbach's alphas (>.7) for the uni‐dimensional measure (confirmed using IRT methods and factor analytic methods). There was moderate evidence in support of test‐retest reliability with correlations >.7. There was moderate evidence for structural validity showing consistent evidence for a one factor solution (using factor analysis). There was moderate evidence for hypothesis testing; the PHQ‐9 correlated strongly with other measures of similar constructs (e.g., the BDI), and support was found for consistent factor structure across time points and sub groups. There was moderate evidence for criterion validity, with acceptable sensitivity and specificity (>.79) in detecting clinical diagnosis of depressive disorder.

***ZDS*.** Two studies were found which explored the measurement properties of the ZDS. There was strong evidence in support of structural validity with a 2‐factor solution. There was weak evidence in support of hypothesis testing, showing significant correlation with other measures of similar constructs (*r* > .61).

***CESD‐R*.** The CESD was recently updated with the CESD‐R to better map onto DSM criteria, so we focused the search on the updated version, as this version will likely be used in future research and clinical practice. Only one study was found which explored the measurement properties of the CESD‐R in general population adults without co‐morbid conditions. Evidence in support of internal consistency was strong with one study rated as excellent methodological quality. Evidence in support of structural validity was strong with the study showing evidence for a single factor solution. Evidence in support of hypothesis testing was moderate, with the study showing acceptable correlations with other measures of similar constructs (*r* > .58).

***MADRS*.** One study was found which explored the measurement properties of the MADRS in general population adults. Evidence for internal consistency was indeterminate with one available study rated as poor, as the uni‐dimensionality of the scale was not checked. Evidence for criterion validity was moderate with evidence for high sensitivity/specificity (>.95) for clinical diagnosis of depression.

## Discussion

Although depression is reported to be highly co‐morbid with ASC, prior to this review it was unknown whether any robust validated tools exist with which to effectively assess depression in adults with ASC. Results reveal a paucity of research with adults with ASC, without co‐morbid intellectual disability, that has used a validated tool to assess depression (12 studies), compared to general population adults (64 studies). Research has also used a smaller range of validated tools to assess depression in those with ASC compared to the general population. Only the BDI‐II, PHQ‐9 and MADRS have been utilized among adults with ASC, whereas research in the general population also frequently uses the HDRS, ZDS, and CESD. Hence, despite depression being a common difficulty among adults with ASC [Ghaziuddin, 2005; Lever & Geurts, 2016], few studies have used a validated depression measure, and no tool is available that has been specifically developed for or comprehensively validated with this group.

Only one study had explored the measurement properties of a validated depression tool—the BDI‐II—in adults with ASC [Gotham, Unruh, & Lord, 2015]. This study provided weak evidence in support of hypothesis testing and criterion validity. However, the study did show an acceptable correlation between scores on the BDI‐II and clinical diagnosis of depression in those with ASC. Hence, the BDI‐II could have utility in assessing depression in adults with ASC. However, larger sample sizes are needed to establish the factor structure, internal consistency, content validity and reliability, to ensure that this measure is appropriate for adults with ASC.

Fourteen studies had explored the measurement properties of the BDI‐II, PHQ‐9, ZDS, CESD‐R and MADRS in general population adults, without co‐morbid conditions. However, none of these studies assessed measurement error or responsiveness to change. Across the studies, several common pitfalls adversely affecting the methodological quality of the studies were identified by the COSMIN checklist. For example, many studies failed to make a number of clear directional hypotheses, making it difficult to interpret the quality of the evidence with respect to hypothesis testing. Many studies failed to report how missing items were dealt with, or check the uni‐dimensionality of the scale before calculating internal consistency, which should be performed separately for each subscale. Despite these common pitfalls in the methodological quality of studies assessing measurement properties of depression measures in general population adults, the BDI‐II and PHQ‐9 were identified as having robust evidence for a range of measurement properties. In particular, both tools show acceptable levels of sensitivity and specificity in detecting clinical diagnosis of depression in general population adult samples. This is reflected in the research literature, as these measures were found to be the most widely used in research from search one. The PHQ‐9 is also recommended in UK NICE guidelines to screen for depression in clinical services.

Results suggest that the BDI‐II and PHQ‐9 could have potential to be adapted for adults with ASC, without co‐morbid intellectual disability. However, it remains unclear whether traditional depression measures can accurately detect depression in those with ASC to a similar degree to those without ASC. For example, questions such as “Trouble falling or staying asleep, or sleeping too much?” and “Moving or speaking so slowly that other people could have noticed?”, overlap with autism symptoms, and may be more likely to be endorsed by those with ASC, even in the absence of depression. In addition to symptomatic overlap between ASC and depression symptoms, the cognitive characteristics of ASC may also affect the usefulness of existing tools to accurately detect depressive symptoms in this group. For example, alexythymia (difficulty articulating one's internal emotional experience) [Bird et al., 2010], and literal interpretation of questions are common in those with ASC [Happé, 1995]. This could affect ability to interpret and answer questions such as “feeling down, depressed or hopeless”, and “I am sad all the time”.

It is also possible that tools developed for the general population are less sensitive in accurately identifying depression in ASC, due to lack of autism‐specific items tailored to the unique presentation of depression in these individuals. For example, loss of absorption in a special interest [Clarke et al., 1989; Gillberg, 1985], agitation, change in sleep pattern or social withdrawal [Ghaziuddin, 2005], have all been identified as potentially unique signs of depression in those with ASC. Recent research has also shown associations between sensory sensitivity and depressive symptoms in those with ASC [Serafini et al., 2017; Bitsika, Sharpley, & Mills, 2016]. Adults with ASC without co‐morbid intellectual disability also frequently report trying to camouflage their symptoms in an attempt to fit into social situations, at great potential cost to their mental health [Lai et al., 2016; Rynkiewicz et al., 2016]. These autism specific traits and behaviors which may be associated with depression in ASC populations have not yet been considered in the context of depression measures. This could mean that current tools may underestimate depression in ASC, as they may not be sensitive to the unique presentation of depression in this group. Future research studies must consider adapting current tools, including such autism specific items.

If it were the case that traditional depression measures are less able to detect depressive symptoms in those with ASC, then we could predict differences in the measurement properties of the instruments between those with and without ASC. For example, traditional depression measures may be less accurate at predicting clinical diagnosis of depression in those with ASC, compared to the general population. The factor structure of these measures may not be the same in those with and without ASC, as the aforementioned items may not be measuring depression per se in those with ASC. Hence, the internal consistency of the measures may also be lower in ASC than general population samples. Conversely, we could predict that autism‐specific measures developed to capture the unique presentation of depression in ASC, would more strongly predict clinical diagnosis of depression in this group compared to traditional measures designed for the general population without ASC. We might also expect the internal consistency of autism‐specific measures to be higher than tools designed for the general population, given that the items should more easily identify symptoms indicating depression in ASC, rather than ASC symptoms more generally. Future research studies should explore the content validity of traditional depression measures in those with ASC, to ensure that items are interpreted appropriately, and relate to the construct to be measured. Additional autism‐specific questions capturing the unique presentation of depression in those with ASC, such as change in social withdrawal, sleep patterns, sensory sensitivity, repetitive behaviors, and loss of interest in a special interest should also be developed and their measurement properties explored in ASC samples. Methods such as cognitive interviewing could be useful in this regard.

This study has a number of strengths as well as limitations. A key strength was using a rigorous method (COSMIN) to systematically identify and evaluate relevant studies. However, following this strict method meant that many studies and some tools were excluded from the analysis. For example, given that a key aim of the current study was to identify robust candidate measures of depression for ASC adults, it was important to focus on tools which include more specific and broader conceptualizations of depression than is feasible in subscales, which typically have a narrower definition of depression and fewer items. This meant that broader mental health assessment tools containing depression subscales such as the SCID were excluded. Subsequently, only one study using the BDI‐II in an ASC sample met criteria to be analyzed by the COSMIN checklist. We also focused the search on studies which included representative general population samples of adults, without co‐morbid conditions, and adults with ASC, without co‐morbid intellectual disability. This ensured that we uncovered evidence relevant to well‐defined representative populations, comparable in terms of age and intellectual functioning. As measures may well operate differently among different populations and contexts, widening the search would likely have produced more mixed results.

In such a new area of research (depression in ASC), it could therefore be argued that adopting such rigorous methods might have lead us to overlook other relevant data which could indicate the usefulness of one tool over another. However, our results are consistent with other COSMIN reviews showing a paucity of research pertaining to the measurement of outcomes in those with ASC [e.g., Hanratty et al., 2015; Wigham & McConachie, 2014], despite a growing number of studies utilizing these tools. This is problematic as many studies which use a tool, do not typically provide adequate evidence regarding the appropriateness or measurement properties of that tool for use in a particular group (e.g., reporting internal consistency without checking the uni‐dimensionality of the scale). This is the primary reason for the development and increased use of COSMIN reviews—to provide clear recommendations for future research and clinical practice on the basis of an internationally standardized rating system. Using a more inclusive approach without clear standardized guidelines would therefore have impeded our ability to make clear evidence based recommendations, or compare our results to the growing number of similar COSMIN reviews in the area of outcome measurement in ASC. Our findings are therefore an important call to action for the research community, to improve the amount and quality of future research regarding the effective identification of depression in ASC, and development of adapted assessment tools for this group.

Our search was also limited by the fact that we focused only on studies in the English language, due to lack of resources to allow translation of articles. Data extraction was also conducted only in part by two independent reviewers. Although COSMIN is a systematic method of searching, compiling and rating evidence with regard to the measurement properties of outcome measures, there is a certain level of subjectivity in rating the quality of the evidence in each article. Therefore, it must be acknowledged that another set of raters may have found slightly different results. However, there was good agreement between raters in the current study (72%), which is similar to previous published work using COSMIN [e.g., Wigham & McConachie, 2014].

In conclusion, this is the first systematic review to use a robust tool (COSMIN) to rate the quality of the evidence with regard to the assessment of depression in adults with and without ASC. There is a lack of evidence regarding the measurement properties of tools in adults with ASC, and no tools which have been developed to assess depression in this group. The BDI‐II and PHQ‐9 both have acceptable evidence in support of their ability to accurately screen for clinical diagnosis of depression in representative general population samples, without co‐morbid conditions. ASC symptoms and cognitive characteristics of this condition could affect the usefulness of these traditional measures to accurately detect depression in ASC populations. The measurement properties of these tools must therefore be explored further in ASC samples, and adaptations to improve their properties considered and tested. Our group are currently undertaking work to explore these questions, in order to ultimately better characterize depression and its measurement in adults with ASC.
